# Relationship between lower-limb muscle strength and frailty among elderly people

**DOI:** 10.1590/S1516-31802012000200006

**Published:** 2012-04-03

**Authors:** Fernanda Sotello Batista, Grace Angelica de Oliveira Gomes, Anita Liberalesso Neri, Maria Elena Guariento, Fernanda Aparecida Cintra, Maria da Luz Rosario de Sousa, Maria José D’Elboux

**Affiliations:** I MSc. Physiotherapist, Municipal Health Department, Santos, São Paulo, Brazil.; II MSc. Physical Educator, Bioscience Institute; UNESP - Universidade Estadual Paulista; Rio Claro Campus; Physical Education Departament; Physical Activity; Health and Sport Laboratory (NAFES); Rio Claro, São Paulo, Brazil.; III MD, PhD. Full Professor, Department of Education, Universidade Estadual de Campinas (Unicamp), Campinas, São Paulo, Brazil.; IV PhD. Professor, Department of Internal Medicine, School of Medical Sciences, Universidade Estadual de Campinas (Unicamp), Campinas, São Paulo, Brazil.; V PhD. Nurse, Department of Nursing, Universidade Estadual de Campinas (Unicamp), Campinas, São Paulo, Brazil.; VI PhD. Professor, Department of Dentistry, Universidade Estadual de Campinas (Unicamp), Piracicaba, São Paulo, Brazil.; VII MD, PhD. Full Professor, Department of Nursing, Universidade Estadual de Campinas (Unicamp), Campinas, São Paulo, Brazil.

**Keywords:** Aged, Muscle strength, Ambulatory care, Frail elderly, Exercise, Idoso, Força muscular, Assistência ambulatorial, Idoso fragilizado, Exercício

## Abstract

**CONTEXT AND OBJECTIVE:**

Sarcopenia is the main factor involved in the development of frailty syndrome. The aims here were to investigate relationships between lower-limb muscle strength and the variables of sex, age and frailty criteria; compare lower-limb muscle strength with each frailty criterion; and assess the power of each criterion for estimating the risk of frailty among elderly outpatients.

**DESIGN AND SETTING:**

Cross-sectional study at the Geriatrics Outpatient Clinic of a university hospital in Campinas.

**METHOD:**

A non-probabilistic convenience sample of 150 elderly people of both sexes who were followed up as outpatients was assessed. Sociodemographic data (sex and age) and physical health data (frailty criteria and the five-times sit-to-stand test) were gathered. Descriptive, comparative and multivariate logistic regression analyses were performed.

**RESULTS:**

The majority of the elderly people (77.3%) were 70 years of age or over, with predominance of females (64.2%) and had a low score for the five-times sit-to-stand test (81.4% scored 0 or 1); 55.3% of the elderly people presented three or more frailty criteria. A significant association was found between lower-limb muscle strength and the variables of age and number of frailty criteria.

**CONCLUSIONS:**

Lower levels of lower-limb muscle strength were associated with advanced age and greater presence of signs of frailty. Moreover, lower-limb muscle strength was also associated with the criteria of reduced walking speed criteria and hand-grip strength.

## RESUMO

**CONTEXTO E OBJETIVO:**

A sarcopenia é o principal fator envolvido no desenvolvimento da síndrome de fragilidade. O objetivo foi investigar a relação entre força muscular de membros inferiores e as variáveis sexo, idade e critérios de fragilidade; comparar a força muscular de membros inferiores com cada critério de fragilidade e verificar seu poder de estimativa do risco para fragilidade em idosos ambulatoriais.

**TIPO DE ESTUDO E LOCAL:**

Estudo transversal no Ambulatório de Geriatria de um hospital universitário de Campinas.

**MÉTODO:**

Foi avaliada uma amostra de conveniência não-probabilística de 150 idosos de ambos os sexos em acompanhamento ambulatorial, com coleta de dados sócio-demográficos (sexo e idade) e de saúde física (critérios de fragilidade e teste de levantar e sentar da cadeira cinco vezes consecutivamente). Foram realizadas análises descritivas, de comparação e de regressão logística multivariada.

**RESULTADOS:**

A maioria dos idosos (77,3%) apresentou idade igual ou superior a 70 anos, com predomínio do sexo feminino (64,0%) e baixo escore no teste de levantar e sentar da cadeira cinco vezes consecutivas (81,4% escore 0 ou 1), 55,3% dos idosos apresentaram três ou mais critérios de fragilidade. Verificou-se associação significativa entre a força muscular de membros inferiores e as variáveis idade e número de critérios de fragilidade.

**CONCLUSÕES:**

Menores níveis de força muscular de membros inferiores estão associados a idade avançada e maior presença de sinais de fragilidade. Além disso, a força muscular de membros inferiores também está associada com os critérios redução da velocidade de marcha e da força de preensão palmar.

## INTRODUCTION

Sarcopenia is the loss of muscle mass and consequent loss of muscle strength that is associated with the aging process. It seems to be the main factor involved in the cycle of the frailty syndrome. Furthermore, sarcopenia also represents the final common pathway in the pathogenesis of frailty syndrome.^1^^,^^2^^,^^3^^,^^4^

Frailty syndrome is a state of vulnerability to stressful factors that cause diminishment of physiological reserves, with subsequent reduction of homeostasis.^5^ Fried and Walston^5^ proposed that the factors involved included neuromuscular abnormalities, deregulation of the neuroendocrine system, dysfunction of the immunological system and a cycle of frailty. The main components would be chronic malnutrition, sarcopenia, decline in physical activities and totally consumed energy.

Sarcopenia is seen as the main contributor to the vicious cycle of frailty. Loss of muscle mass is responsible for reductions in the underlying metabolic rate and in physical activity, and these changes may lead to deregulation of energy production and consumption, thereby resulting in malnutrition.^2^^,^^3^^,^^4^^,^^5^^,^^6^ In addition, the literature indicates that decreased muscle strength is associated with high levels of interleukin-6 and tumor necrosis factor alpha, and with low levels of growth hormones, growth factors similar to isoform insulin I and sexual hormones.^1^^,^^6^^,^^7^

Fried et al.^8^ established five criteria for diagnosing frailty: involuntary weight loss, decreased walking speed, low levels of physical activity, exhaustion and decreased grip strength. Subjects are considered pre-frail in the presence of one or two frailty criteria and frail if they present three or more criteria.

Although the phenotype elaborated by Fried et al.^8^ is frequently used in research internationally, some researchers have proposed a more simplified index to detect frailty, with the objective of facilitating clinical practice and identifying individuals at risk.^9^^,^^10^^,^^11^ Purser et al.^9^ evaluated 309 elderly people to investigate whether single measurements of physical performance, such as walking speed, five-times sit-to-stand test and grip strength might be good frailty indicators. They concluded that all of these measures, individually, were able to identify frailty.

The five-times sit-to-stand test is simple, fast, inexpensive and reproducible. Not much space or special equipment is required to perform it: a chair and a chronometer are enough. The five-times sit-to-stand test has been used in some studies^12^^,^^13^^,^^14^ as an indicator of lower-limb muscle strength, and it is measured as the time taken to complete five repetitions of the sit/stand movement on an armless chair.

## OBJECTIVE

The aims of the present study were: (1) to investigate relationships between lower-limb muscle strength and the variables of sex, age and frailty criteria among elderly outpatients; (2) to compare the lower-limb muscle strength of these elderly individuals with each frailty criterion; and (3) to assess the power of each criterion for estimating the risk of frailty among these individuals.

## METHOD

This was a cross-sectional study, extracted from a larger study on frailty developed in the Geriatrics Outpatient Clinic of a university hospital in Campinas, State of São Paulo, Brazil. It was approved by the institution’s Research Ethics Committee, as decision no. 240/2003. The elderly people treated in the outpatient clinic had a minimum age of 80 years or a minimum age of 60 years with functional impairment.

The present study used a non-probabilistic convenience sample of 150 subjects between October 2004 and October 2006. The subjects met the following inclusion criteria: agreeing to participate in the study, signing a free and informed consent statement and being in a cognitive condition for oral communication to be established so that an interview could be conducted and the Mini-Mental State Examination could be applied, as prescribed by Bertolucci et al.^15^

As many older adults as possible per day were approached to inquire about their willingness to take part in the study, their availability for an interview and their compatibility with the inclusion criteria. We were able to interview two older adults a day.

- To identify frailty, the criteria of Fried et al.^8^ were used with some adaptations, as follows:
- Involuntary weight loss of more than 4.5 kilograms or 10% of body weight over the last year.
- Exhaustion: Self-reported fatigue was investigated through two questions: “Did you feel that you had to make an effort to accomplish your habitual tasks?” and “Were you unable to do your things?” These were taken from the depression tracking scale developed by Radloff^16^ at the Center for Epidemiologic Studies and validated in Brazil for the elderly population by Batistoni et al.^17^ In the case of an affirmative answer for a period of three or more days of the previous week, the subject was graded as positive for exhaustion.
- Decreased walking speed: The time taken to walk a distance of 4.0 meters one way and 4.0 meters to come back was measured using a chronometer, taking the best time for this course, adjusted according to sex and height. For men of heights < 1.73 and > 1.73 meters respectively, times of > 7 seconds and > 6 seconds were considered positive. For women of heights < 1.59 and > 1.59 meters respectively, times of > 7 seconds and > 6 seconds were considered positive.
- Muscle weakness: This was evaluated by means of a grip dynamometer. The individual under evaluation was positioned standing upright with the arms along the body, except for wheelchair users, who did the test in the seated position. The highest value was taken from three measurements of grip strength, with intervals of approximately five minutes between them, adjusted for sex and body mass index. Men were graded positive for muscle weakness when their grip strength was < 29.0 kgf for body mass index < 24.0 kg/m^2^; < 30.0 kgf for body mass index of 24.1 to 26.0; and < 32.0 kgf for body mass index > 28.0. For women, the values were < 17.0 kgf for body mass index < 23.0; < 17.3 kgf for body mass index of 23.1 to 26.0; < 18.0 kgf for body mass index of 26.1 to 29.0; and < 21.0 kgf for body mass index > 29.0.

Low level of physical activity: This was graded positive for elderly people who were inactive or who performed physical activities < 2 times a week.

After the evaluation of frailty criteria, two groups were obtained: one group with one or two criteria (considered pre-frail) and the other with three or more criteria (considered frail), as suggested by Fried et al.^8^ All of the subjects in this study presented at least one of the criteria.

To evaluate lower-limb muscle strength, the five-times sit-to-stand test was used. This forms part of the Short Physical Performance Battery instrument, which was proposed by Guralnik et al.^12^ and adapted to the Brazilian Portuguese language by Nakano.^18^ The test was undertaken using an armless chair that was 46 centimeters high from its seat to the floor. The individual under evaluation sat on the chair with arms folded across the chest. Points were awarded according to the time needed to complete the test. Zero was attributed when the elderly individual was unable to perform the test or required > 60 seconds to complete it; one point if the time needed was > 16.7 seconds; two points if the time was between 13.70 and 16.69 seconds; three points if the time was between 11.20 and 13.69 seconds; and 4 points if the time was < 11.19 seconds. Scores 3 and 4 for the lower-limb muscle strength test were grouped together because of the small number of subjects with each score (n = 8 and n = 6, respectively).

For the present study, the following variables were extracted from the database of a larger study on frailty: personal identification, sociodemographic data, physical health, anthropometry, mobility and flexibility (walking speed and lower-limb muscle strength), physical activity, mini-mental state examination and state of depression (evaluation of the frailty criterion that was identified as exhaustion).

Descriptive statistical analyses were performed to draw up tables with absolute frequencies (n) and relative frequencies (percentages or proportions of answers) of the categorical variables. To compare the categorical variables between groups, the chi-square test was used, or when necessary (when the expected values were smaller than 5), Fisher’s exact test was used. Multivariate logistic regression analysis was used when it was foreseen that classification in the category y = 1 was likely. P was used to analyze the estimated risk that three to five frailty criteria might be present, with regard to lower-limb muscle strength and other variables of interest. Elderly people with one or two frailty criteria were compared with those presenting three or more frailty criteria. The significance level adopted was 5% (P < 0.05).

## RESULTS

Table 1 shows that most of the elderly people (77.3%) were aged 70 years or over, with predominance of female (64.2%), married (46.4%), retired (85.3%) and literate (64.9%) subjects. In this sample, 122 elderly individuals (81.4%) presented low scores for lower-limb muscle strength (0 and 1); 44.7% presented one or two frailty criteria (considered pre-frail), while 55.3% presented three or more criteria (considered frail).

Comparison between the lower-limb muscle strength scores and the variables of interest showed that were significant associations with age (P = 0.044) and the number of frailty criteria (P = 0.010), as presented in Table 2. Low scores (0 and 1) for lower-limb muscle strength correlated with greater numbers of elderly people aged 70 years or over and with greater numbers of frailty criteria.

The comparisons between lower-limb muscle strength and each of the frailty criteria (involuntary weight loss, exhaustion, decreased walking speed, low level of physical activity and reduced grip strength) are presented in Table 3. Lower-limb muscle strength was significantly associated with the criteria of decreased walking speed (P < 0.001) and reduced grip strength (P = 0.045), thus showing that the elderly people with scores for the criteria of decreased walking speed and reduced grip strength presented lower scores for lower-limb muscle strength.

Multivariate logistic regression analysis was carried out to study the risk of presenting frailty (three or more criteria) in relation to lower-limb muscle strength, with comparison between pre-frail and frail elderly people, as shown in Table 4.

The odds ratio for the elderly people to be frail was 4.8 times higher among those who scored zero points for lower-limb muscle strength and 3.5 times higher among those who scored one point.

Regarding the age variable, the elderly people aged 70 to 79 years presented an odds ratio that was 3.82 times higher, and for those aged 80 or over, the odds ratio was 2.83 times higher.

Female sex corresponded to an odds ratio for frailty that was 1.85 times higher than shown by male sex. The odds ratio for frailty was highest among elderly females aged 70 years or over who also had low scores for lower-limb muscle strength.

**Table 1.** Description of sociodemographic characteristics and health of the subjects studied (n = 150)

Variables	Category	n (%)
Age (in years)	60 - 69	34 (22.7)
	70 - 79	65 (43.5)
	≥ 80	51 (33.8)
Sex	Female	96 (64.2)
	Male	54 (35.8)
Marital status	Married	70 (46.4)
	Widowed	64 (42.7)
	Divorced	8 (5.3)
	Single	8 (5.3)
Literate	Yes	97 (64.9)
	No	53 (35.1)
Retired	Yes	128 (85.3)
	No	22 (14.7)
Lower-limb muscle strength (score)	0	26 (17.4)
	1	96 (64.0)
	2	14 (9.3)
	3 - 4	14 (9.3)
No. of frailty criteria	1 - 2	67 (44.7)
	≥ 3	83 (55.3)

**Table 2.** Comparison between the lower-limb muscle strength and the sex, age and frailty criteria of the elderly individuals studied (n = 150)

Variable	Category	n (%)	Lower-limb muscle strength				P-value
			0	1	2	3-4	
			n (%)	n (%)	n (%)	n (%)	
Sex	Male	54 (36.0)	6 (23.1)	38 (39.6)	7 (50.0)	3 (21.4)	0.179^*^
	Female	96 (64.0)	20 (76.9)	58 (60.4)	7 (50.0)	11 (78.6)	
Age (years)	60 - 69	34 (22.7)	4 (15.4)	18 (18.7)	4 (28.6)	8 (57.1)	0.044^†^
	70 - 79	65 (43.3)	9 (34.6)	45 (46.9)	7 (50.0)	4 (28.6)	
	≥ 80	51 (34.0)	13 (50.0)	33 (34.4)	3 (21.4)	2 (14.3)	
No. of frailty criteria	1 - 2	67 (44.7)	8 (30.8)	39 (40.6)	9 (64.3)	11 (78.6)	0.010^*^
	≥ 3	83 (55.3)	18 (69.2)	57 (59.4)	5 (35.7)	3 (21.4)	

^*^Chi-square test; ^†^ Fisher’s exact test.

**Table 3.** Comparison of subjects’ scores in the lower-limb muscle strength test, according to the presence of each frailty criterion

Variable	n (%)	Lower-limb muscle strength				P-value
		0	1	2	3-4	
		n (%)	n (%)	n (%)	n (%)	
Low level of physical activity	120	22	75	11	12	0.913*
	(100)	(18.3)	(62.5)	(9.2)	(10.0)	
Involuntary weight loss	65	11	43	6	5	0.929†
	(100)	(16.9)	(66.2)	(9.2)	(7.7)	
Reduced grip strength	87	14	63	5	5	0.045*
	(100)	(16.1)	(72.4)	(5.7)	(5.7)	
Decreased walking speed	82	24	49	7	2	< 0.001*
	(100)	(29.3)	(59.8)	(8.5)	(2.4)	
Fatigue	40	9	25	2	4	0.555†
	(100)	(22.5)	(62.5)	(5.0)	(10.0)	

^*^Chi-square test; ^†^ Fisher’s exact test.

**Table 4.** Multivariate logistic regression analysis for the risk of three to five frailty criteria in the sample studied (n = 150)

Variables	Categories	P-value	OR	95% CI
Age(years)	60-69 (ref)		1.00	---
	70-79	0.002	4.82	1.80 - 12.91
	≥ 80	0.009	3.83	1.39 - 10.51
Sex	Male (ref)		1.00	---
	Female	0.007	2.85	1.33 - 6.11
Lower-limb muscle strength	3 or 4 points (ref)		1.00	---
	2 points	0.648	1.87	0.31 - 11.29
	1 point	0.038	4.50	1.07 - 18.91
	0 points	0.031	5.89	1.17 - 29.71

C = 0.732 (model accuracy); OR = odds ratio for frailty; 95% CI = 95% confidence interval for odds ratio

## DISCUSSION

The low performance in the five-times sit-to-stand test presented by most of the elderly subjects and the presence of at least one frailty criterion in all the subjects evaluated may be related to the profile of the elderly individuals attended at the Geriatric Outpatient Clinic that was used as the study field. These patients were either more than 80 years of age or more than 60 years of age if they had some degree of dependence.

In the study sample, the majority presented a low score in the test on lower-limb muscle strength. This result was similar to the findings of Mazzà et al.,^19^ who observed that there was a higher percentage of elderly outpatients with scores of 0 and 1 in the five-times sit-to-stand test (27.5% of the subjects for each of these scores). However, the present study differed from the studies by Barbosa et al.^20^ on elderly people within the community and Ferreira et al.^21^ on elderly outpatients, who both found that the majority of their subjects had higher scores in this test. On the other hand, differing from the present study, one of the criteria used by these authors was a requirement that the subjects should be in a condition to walk.

In relation to lower-limb muscle strength, there was a significant association with lower scores among individuals aged 70 years or over. This finding is consistent with other research.^20^^,^^22^^,^^23^ In evaluations on elderly residents within the community, Barbosa et al.,^20^ Lord et al.^22^ and Forrest et al.^23^ found a significant correlation (P < 0.001) between the five-times sit-to-stand test and age, such that the execution time for the test became longer with increasing age.

The results from the present study did not indicate any difference in the five-times sit-to-stand test between the sexes. This corroborated the results obtained by Singh et al.^24^ among institutionalized elderly people, Mazza et al.^19^ among elderly outpatients and Lord et al.^22^ among elderly people living in the community. On the other hand, studies by Barbosa et al.^20^ among elderly people within the community and Aslan et al.^25^ among elderly outpatients obtained significant differences (P < 0.005) in the five-times sit-to-stand test between the sexes, with higher scores for males. Barbosa et al.^20^ explained this in terms of the greater presence of chronic sicknesses and obesity among the women in their study population and Aslan et al.^25^ suggested that new studies should be conducted to clarify their findings.

In addition, low test scores for lower-limb muscle strength correlated with greater concentration of elderly people presenting three or more frailty criteria. In the literature consulted for this study, it was seen that few studies make reference to the relationship between lower-limb muscle strength, as measured by the five-times sit-to-stand test and frailty criteria, thereby making it difficult to compare these findings. However, the results from the study by Ottenbacher et al.^26^ showed that lower-limb muscle strength measured through manual dynamometry was a strong predictor of frailty. The study by Boxer et al.^27^ is noteworthy, since it found that the six-minute walk test could discriminate between pre-frail and non-frail elderly people suffering from heart failure.

Furthermore, comparison of lower-limb muscle strength with each of the frailty criterion allowed the observation that elderly people with lower scores presented positive scores for the walking-speed and grip-strength criteria. These results are similar to those obtained by Barbosa et al.,^20^ Singh et al.^24^ and Ottenbacher et al.,^26^ who found correlations between these variables. However, Ottenbacher et al.^26^ used a different test to measure lower-limb muscle strength, instead of the five-times sit-to-stand test.

The present study enabled the finding that female individuals aged 70 years and over whose lower-limb muscle strength scores were 0 or 1 presented a higher risk of frailty. The results from the studies by Fried et al.,^8^ Woods et al.,^28^ Ávila-Funes et al.^29^ and Ottenbacher et al.^30^ demonstrated significant correlations (P < 0.05) between female sex, advanced age and frailty. Ottenbacher et al.^30^ only found an association between female sex and frailty, while Purser et al.^9^ also found that the five-times sit-to-stand test was a strong predictor of frailty. These findings show the relevance and applicability of this study, which makes it simpler to identify frailty risk groups through an easily applied lower-limb muscle strength test. It has also been widely reported in the literature^31^^,^^32^^,^^33^^,^^34^^,^^35^ that, with exercise practice guided by qualified professionals, the loss of muscle strength due to aging is potentially reversible.

In the present study, the five-times sit-to-stand test was found to be sufficient for defining the loss of muscle mass. It presented an association with two out of the five frailty criteria (decreased walking speed and reduced grip strength) that are used to detect frailty syndrome. The simplicity of performing this test and comparisons between this test and the walking speed and handgrip strength tests make it easier and quicker to evaluate elderly individuals, thereby allowing its use in categorizing outpatients according to their risk of frailty.

## CONCLUSION

The results obtained in this study reveal that diminished lower-limb muscle strength is associated with a higher risk of frailty. Diminished lower-limb muscle strength was observed among individuals of greater age (³ 70 years) and larger numbers of frailty criteria (³ 3 criteria), without differences between the sexes.

There are some limitations to the present study. Firstly, there was no group of elderly people without frailty criteria (i.e. non-frail individuals). This is possibly related to the profile of elderly patients attended by this Geriatric Outpatient Clinic. Nonetheless, it was still possible to prove the discrimination potential of the five-times sit-to-stand test. It was possible to identify significant differences in lower-limb muscle strength between the groups with one or two frailty criteria and with three or more criteria.

It also needs to be borne in mind that the elderly people of this sample presented particular characteristics and, therefore, the results should not be extrapolated to the general population. Furthermore, according to the results, using the lower-limb muscle strength test instead of the handgrip strength and walking speed test may be enough for effective outpatient frailty screening. Thus, decreased lower-limb muscle strength in elderly patients should be evaluated thoroughly and is an important risk factor for frailty syndrome.

## References

[1] Silva TAA, Frisoli A, Pinheiro MM, Szejnfeld VL (2006). Sarcopenia associada ao envelhecimento: aspectos etiológicos e opções terapêuticas. (Sarcopenia and aging: etiological aspects and therapeutic options). Rev Bras Reumatol.

[2] Cesari M, Leeuwenburgh C, Lauretani F (2006). Frailty syndrome and skeletal muscle: results from the Invecchiare in Chianti study. Am J Clin Nutr.

[3] Walston J, Hadley EC, Ferrucci L (2006). Research agenda for frailty in older adults: toward a better understanding of physiology and etiology: summary from the American Geriatrics Society/National Institute on Aging Research Conference on Frailty in Older Adults. J Am Geriatr Soc.

[4] Abate M, Di Iorio A, Di Renzo D (2007). Frailty in the elderly: the physical dimension. Eura Medicophys.

[5] Fried LP, Walston J, Hazzard WR, Blass JP, Halter JB, Ouslander JG, Tinetti ME (2003). Frailty and failure to thrive. Principles of geriatric medicine and gerontology.

[6] Macedo C, Gazzola JM, Najas M (2008). Síndrome da fragilidade no idoso: importância da fisioterapia. (Frailty syndrome in elderly patients: the importance of physiotherapy). Arq Bras Ciênc Saude.

[7] Edström E, Altun M, Bergman E (2007). Factors contributing to neuromuscular impairment and sarcopenia during aging. Physiol Behav.

[8] Fried LP, Tangen CM, Walston J (2001). Frailty in older adults: evidence for a phenotype. J Gerontol A Biol Sci Med Sci.

[9] Purser JL, Kuchibhatla MN, Fillenbaum GG (2006). Identifying frailty in hospitalized older adults with significant coronary artery disease. J Am Geriatr Soc.

[10] Ensrud KE, Ewing SK, Taylor BC (2008). Comparison of 2 frailty indexes for prediction of falls, disability, fractures, and death in older women. Arch Intern Med.

[11] Ensrud KE, Ewing SK, Cawthon PM (2009). A comparison of frailty indexes for the prediction of falls, disability, fractures, and mortality in older men. J Am Geriatr Soc.

[12] Guralnik JM, Simonsick EM, Ferrucci L (1994). A short physical performance battery assessing lower extremity function: association with self-reported disability and prediction of mortality and nursing home admission. J Gerontol.

[13] McCarthy EK, Horvat MA, Holtsberg PA, Wisenbaker JM (2004). Repeated chair stands as a measure of lower limb strength in sexagenarian women. J Gerontol A Biol Sci Med Sci.

[14] Takai Y, Ohta M, Akagi R (2009). Sit-to-stand test to evaluate knee extensor muscle size and strength in the elderly: a novel approach. J Physiol Anthropol.

[15] Bertolucci PH, Brucki SM, Campacci SR, Juliano Y (1994). O Mini-Exame do Estado Mental em uma população geral. Impacto da escolaridade (The Mini-Mental State Examination in a general population: impact of educational status). Arq Neuropsiquiatr.

[16] Radloff LS (1977). The CES-D Scale: A Self-Report Depression Scale for Research in the General Population. Applied Psychological Measurement.

[17] Batistoni SS, Neri AL, Cupertino AP (2007). Validade da escala de depressão do Center for Epidemiological Studies entre idosos brasileiros. (Validity of the Center for Epidemiological Studies Depression Scale among Brazilian elderly). Rev Saude Publica.

[18] Nakano MM (2007). Versão brasileira da Short Physical Performance Battery? SPPB: adaptação cultural e estudo da confiabilidade (Dissertation) (Brazilian version of the Short Physical Performance Battery - SPPB: cross-cultural adaptation and reliability study).

[19] Mazzà C, Benvenuti F, Bimbi C, Stanhope SJ (2004). Association between subject funcional status, seat height, and movement strategy in sit-to-stand performance. J Am Geriatr Soc.

[20] Barbosa AR, Souza JM, Lebrão ML, Laurenti R, Marucci Mde F (2005). Functional limitations of Brazilian elderly by age and gender differences: data from SABE Survey. Cad Saude Publica.

[21] Ferreira FFP, Izzo H, Jacob W (2007). Impacto da capacidade física na saúde percebida entre idosos em velhice avançada. Saúde Coletiva.

[22] Lord SR, Murray SM, Chapman K, Munro B, Tiedemann A (2002). Sit-to-stand performance depends on sensation, speed, balance and psychological status in addition to strength in older people. J Gerontol A Biol Sci Med Sci.

[23] Forrest KY, Zmuda JM, Cauley JA (2006). Correlates of decline in lower extremity performance in older women: A 10-year follow-up study. J Gerontol A Biol Sci Med Sci.

[24] Singh AS, Chin A Paw MJ, Bosscher RJ, van Mechelen W (2006). Cross-sectional relationship between physical fitness components and functional performance in older persons living in long-term care facilities. BMC Geriatr.

[25] Aslan UB, Cavlak U, Yagci N, Akdag B (2008). Balance performance, aging and falling: a comparative study based on a Turkish sample. Arch Gerontol Geriatr.

[26] Ottenbacher KJ, Ostir GV, Peek MK (2005). Frailty in older Mexican Americans. J Am Geriatr Soc.

[27] Boxer RS, Wang Z, Walsh SJ, Hager D, Kenny AM (2008). The utility of the 6-minute walk test as a measure of frailty in older adults with heart failure. Am J Geriatr Cardiol.

[28] Woods NF, LaCroix AZ, Gray SL (2005). Frailty: emergence and consequences in women aged 65 and older in the Women’s Health Initiative Observational Study. J Am Geriatr Soc.

[29] Avila-Funes JA, Helmer C, Amieva H (2008). Frailty among community-dwelling elderly people in France: the three-city study. J Gerontol A Biol Sci Med Sci.

[30] Ottenbacher KJ, Graham JE, Al Snih S (2009). Mexican Americans and frailty: findings from the Hispanic established populations epidemiologic studies of the elderly. Am J Public Health.

[31] Borst SE (2004). Interventions for sarcopenia and muscle weakness in older people. Age Ageing.

[32] Latham NK, Bennett DA, Stretton CM, Anderson CS (2004). Systematic review of progressive resistance strength training in older adults. J Gerontol A Biol Sci Med Sci.

[33] Aveiro MC, Granito RN, Navega MT, Driusso P, Oishi J (2006). Influence of a physical training program on muscle strength, balance and gait velocity among women with osteoporosis. Rev Bras Fisioter.

[34] Candeloro JM, Caromano FA (2007). Efeito de um programa de hidroterapia na flexibilidade e na força muscular de idosas (Effect of a hydrotherapy program on flexibility and muscle strength in elderly women). Rev Bras Fisioter.

[35] Eyigor S, Karapolat H, Durmaz B (2007). Effects of a group-based exercise program on the physical performance, muscle strength and quality of life in older women. Arch Gerontol Geriatr.

